# Opioid substitution treatment with sublingual buprenorphine in Manipur and Nagaland in Northeast India: what has been established needs to be continued and expanded

**DOI:** 10.1186/1477-7517-6-4

**Published:** 2009-02-26

**Authors:** M Suresh Kumar, Richard D Natale, B Langkham, Charan Sharma, Rachel Kabi, Gordon Mortimore

**Affiliations:** 1 Chennai and National Institute of Epidemiology (Indian Council of Medical Research), Chennai, India; 2 DFID PMO, New Delhi, 110016, India; 3 Australia International Health Institute (AIHI), University of Melbourne, Melbourne, Australia; 4 Emmanuel Hospital Association (EHA), New Delhi, India; 5 HAARP Regional Technical Coordination Unit, Chamnan Phenjati Building, 2nd Floor 65/32 Rama 9 Road, Huay Kwang, Bangkok 10310, Thailand

## Abstract

Manipur and Nagaland in northeast India report an antenatal HIV prevalence of > 1% and the current HIV prevalence among injecting drug users is 24% and 4.5% respectively. Through support from DFID's Challenge Fund, Emmanuel Hospital Association (EHA) established thirteen drop-in-centres across the two states to deliver opioid substitution treatment with sublingual buprenorphine for 1200 injecting drug users. Within a short span of time the treatment has been found to be attractive to the clients and currently 1248 injecting opioid users are receiving opioid substitution treatment. The project is acceptable to the drug users, the families, the communities, religious as well as the militant groups. The treatment centres operate all days of the week, have trained staff members, utilize standardized protocols and ensure a strict supervised delivery system to prevent illicit diversion of buprenorphine. The drug users receiving the substitution treatment are referred to HIV voluntary counselling and testing. As this treatment has the potential to change HIV related risk behaviours, what has been established in the two states needs to be continued and expanded with the support from the Government of India.

## Findings

In the early 1980s injecting heroin became popular in northeast India, sharing border with Myanmar and since then injecting has diffused extensively into many states of northeast India [1,2]. Recent size estimation data show that injecting drug users (IDUs) could constitute 1·9–2·7% of the adult population in Manipur and Nagaland [3]. Ever since HIV was first reported among IDUs in Manipur, HIV infection has rapidly diffused and escalated among the IDUs in the region [4-6]. The HIV prevalence among IDUs during the 2002, 2003, 2004 and 2005 sero sentinel surveillance was estimated at 39%, 24.5%, 21%, 24% in Manipur and 10%, 8.4%, 3.2%, 4.5% in Nagaland respectively [7]. Sexual transmission of HIV from IDUs to their non-injecting wives has occurred in Manipur [8]. Manipur and Nagaland in northeast India are among the high prevalent HIV states in India and the antenatal HIV prevalence in both states is > 1% [7,9]. The community outreach based interventions for IDUs have been established since early 1990s [10]. Harm reduction interventions have been advocated in the northeastern states to deal with the increasing HIV epidemic among IDUs and they have been driven by community based organizations run by former drug users [11-13]. The targeted interventions supported by the State AIDS Control Societies focus on community outreach based education; distribution of needles and syringes and condoms; and, referral for voluntary confidential, counselling and testing (VCCT). Methadone is not available in India for clinical use since the time it was taken off the Indian pharmacopoeia in 1982. Sublingual buprenorphine is licensed in India for treatment of opioid dependence by drug abuse treatment centres since 1999 [14]. Though an opioid substitution treatment (OST) with sublingual buprenorphine was established and operated in Imphal, Manipur by a non-Governmental organization (NGO) during 1999–2002 targeting about 250 IDUs, the lessons learnt from the project were not documented in detail.

It is evident from the rapid assessments carried out in Manipur that injecting drug users preferred OST [15]. Having understood the need from the drug using populations in the Northeastern States of India, the Emmanuel Hospital Association (EHA) of India successfully applied for a grant from the DFID Challenge Fund [16] in order to establish OST for opioid injectors in the states of Manipur and Nagaland. EHA identified NGOs working with drug users in Manipur (n = 7) and Nagaland (n = 4) to establish OST in nine drop-in-centres (DICs) in Manipur and four DICs in Nagaland to cover a target of 1200 IDUs. Five months after the initiation of the opioid substitution treatment, a mid-term evaluation was carried out by two external consultants during the month of October 2006. The methods of evaluation included personal visits to the DICs for observation, in-depth interviews with the staff of the DICs and focus group discussions (FGDs) with the clients attending the services as well as review of all relevant documentation.

In addition, two workshops were held in which the program managers presented the findings of the project based on a standardized format provided to them earlier. During the workshop, small group discussions were held with the various groups of program managers, health care workers and outreach workers in order to identify the challenges and possible future directions.

Table 1 describes some of the findings of the evaluation. In all the sites the number of drug users attending the services exceeded the targeted number. In-depth interviews indicate that within a few days of initiation of the treatment project, the treatment slots were filled. A total of 451 opioid users are on the waitlist as of 30^th ^Sept 2006. The primary drugs of use in the drug users seeking substitution treatment in Manipur are: heroin (560/844; 66.4%); a combination of heroin and dextropropoxyphene (207/844; 24.5%) and dextropropoxyphene alone (77/844; 9.1%). In Nagaland, the primary drugs of use are: a combination of the adulterated heroin [brown sugar] and dextropropoxyphene (197/404; 48.8%), dextropropoxyphene alone (160/404; 39.6%), followed by brown sugar (47/404; 11.6%). The number of women drug users in treatment is low (102/1248; 8.2%) and only one of the thirteen DICs is targeting women drug users and their regular sex partners. A total of 446 opioid users (446/1353; 33%) in Manipur and 106 opioid users (106/537; 19.7%) in Nagaland dropped out after commencing treatment. The reasons for drop-out identified through FGDs are:

**Table 1:** Opioid users under treatment with sublingual buprenorphine in the northeastern states of Manipur and Nagaland, India

**Name of NGO**	**Target**	**Waitlist**	**Currently under treatment**		**Primary drug of use**			**HIV testing and ART treatment**		
			
			**Total**	**Females (%)**	**Heroin or brown sugar**	**Dextropropoxyphene**	**Combination**	**HIV status known**	**Ref for VCCT**	**Ref for ART**
**Manipur**										

Care Foundation (Imphal)	100	51	91	3 (3.3%)	80 (87.9%)	11 (12.1%)	0 (0%)	49 (53.8%)	56 (61.5%)^‡^	7 (7.7%)

Care Foundation (Nambol)	75	0	60	1 (1.7%)	31(51.7%)	25 (41.7%)	4 (6.7%)	9 (15%)	35 (58.3%)	0 (0%)

DPU	75	25	94	3 (3.2%)	63 (67%)	0 (0%)	31 (33%)	24 (25.5%)	24 (25.5%)	2 (2.1%)

MNP+	100	150	102	0 (0%)	80 (78.4%)	7 (68.6%)	15 (14.7%)	65 (63.7%)	32 (31.4%)	9 (8.8%)

Ramungo Library	75	0	77	0 (0%)	4 (5.2%)	4 (5.2%)	69 (89.6%)	6 (7.8%)	21(27.3%)	2 (2.6%)

SASO (East)	100	30	100	0 (0%)	61 (61%)	4 (4%)	35 (35%)	32 (32%)	15 (15%)	1 (1%)

SASO (West)	100	27	100	2 (2%)	72 (72%)	10 (10%)	18 (18%)	26 (26%)	19 (19%)	6 (6%)

Sahara	100	0	110	7 (6.4%)	101(91.8%)	4 (3.7%)	5 (4.5%)	44 (40%)	35 (31.8%)	23 ((20.9%)

SHALOM	100	0	110	7 (6.4%)	68 (61.8%)	12 (10.9%)	30 (27.3%)	2 (1.8%)	23 (20.9%)	9 (8.2%)

**Manipur Total**	**825**	**283**	**844**	**23 (2.7%)**	**560 (66.4%)**	**77 (9.1%)**	**207 (24.5%)**	**257 (30.5%)**	**260 (30.8%)**	**59 (7%)**

**Nagaland**										

Bethesda (IDU)	100	168	114	0 (0%)	0 (0%)	1 (0.9%)	113 (99.1%)	3 (2.6%)	15 (13.2%)	0 (0%)

Bethesda (IDUSW)	100	0	106	79 (73.1%)	16 (15.1%)	12 (11.3%)	78 (73.6%)	13 (12.3%)	12 (11.3%)	0 (0%)

Bethesda- Pfutsero	75	0	60	0 (0%)	0 (0%)	54 (90%)	6 (10%)	0 (0%)	0 (0%)	0 (0%)

Kripa Foundation	100	0	124	0 (0%)	31(25%)	93 (75%)	0 (0%)	0 (0%)	11 (8.9%)	0 (0%)

**Nagaland Total**	**375**	**168**	**404**	**79 (19.6%)**	**47 (11.6%)**	**160 (39.6%)**	**197 (48.8%)**	**16 (4%)**	**38 (9.4%)**	**0 **(0%)

**Grand Total**	**1200**	**451**	**1248**	**102 (8.2%)**	**607 (48.6%)**	**237 (19%)**	**404 (32.4%)**	**273 (21.9%)**	**298 (23.9%)**	**59 (4.7%)**

• Distance of the DICs from the residence of the drug users,

• Difficulty in follow-up due to either the wrong addresses provided by the drug users or the limited number of outreach staff in the projects.

The treatment is delivered through a protocol adapted from the guidelines developed by UNODC ROSA [17]. Doctors, either full time or part time are available in all the DICs. The average maintenance dose is 4–8 mg in 12 of the 13 DICs; in one centre in Nagaland, the average dose is 12 mg. All the thirteen DICs provide sublingual buprenorphine tablets strictly under supervision. The medicine is crushed and administered underneath the tongue by the health care worker and the patients are asked to stay on the premises for 15 minutes after administration of the drug. All DICs operate 7 days a week and the dispensing time is from morning until afternoon in eight DICs, whilst the remaining five operate from morning through to the evening. Six of the nine DICs in Manipur allow take home doses that are not exceeding three days save for in exceptional cases (e.g., visit to village to attend a death or marriage of a close relative); the take home doses are given to a family member.

The other services provided to all drug users in the DICs are: condoms, STI treatment, wound care and primary medical care. A total of 257 (257/844; 30.5%) and 16 (16/404; 4%) drug users on substitution treatment in Manipur and Nagaland respectively, were aware of their HIV status at the time of recruitment. Since initiating buprenorphine treatment, 260 drug users (260/844; 30.8%) in Manipur and 38 drug users (38/404; 9.4%) in Nagaland have been referred for VCCT. Of the drug users receiving treatment, 4.7% (59/1248) have been referred for Antiretroviral Treatment (ART). The other referrals that are offered to the drug users include: assessing liver function tests, STI treatment and TB treatment. A total of 109 drug users (109/1248; 8.7%) have been referred for abstinence oriented treatment.

The majority of staff members (96/110; 87.2%) are trained in OST. All the treatment centres have facilitated the formation of support groups and many of the regular clients serve as peer volunteers and help with patient education, DIC maintenance and cleanliness of the surroundings. All the DICs have carried out advocacy with the community, particularly the neighborhood; two DICs in Nagaland and one DIC in Manipur have advocated with underground militants and obtained support from them to carry out the project without any interference. Two DICs each in Manipur and Nagaland have carried out advocacy with the police. Three DICs have advocated with policy makers and health professionals.

Within a short span of time, all DICs have recruited opioid users without the active help of the outreach workers and inducted them into OST. The treatment is attractive to clients as indicated by the wait-list in all of the DICs. OST is acceptable to all stakeholders – drug users, families of drug users, religious leaders, law enforcement and the underground militants, and this a key factor for enabling the DICs to operate without a hitch in all the places. The trained staff members, utilization of standardized protocols by the medical doctors and the help of the peer volunteers facilitate a good process for the delivery of OST. A strict supervised delivery system ensures that there is no illicit diversion of the drug. While on OST, the drug users are able to utilize HIV voluntary counselling and testing services. The data on behavioural changes is being collected and will be analysed at the end of a year following the establishment of OST. The project must address issues relating to retention and the further improvement of retention rates. Retention rates can be improved by providing improved access to transportation, encouraging the drug-users to provide correct personal information and by increasing the number of outreach workers in each DIC to do follow-up work.

The primary concern expressed by the drug users and the staff members is the issue of sustainability. Given that the project is attractive, acceptable and capable of providing a range of prevention and care services for the drug users while on OST, it is vital that there be a continuity of the services that have been established. While the National AIDS Control Organization (NACO) in India is contemplating scaling up prevention and care services for IDUs in the next phase of programme implementation, lessons learnt from the OST projects in the two states is of immense value. The projects have demonstrated that community based organizations can establish OST that can serve the drug users in user-friendly settings that can offer a continuum of prevention and care services through effective linkages and referrals to existing health services. What is required is policy advocacy to ensure that the drug users have access to the drugs like buprenorphine, an essential drug listed by the WHO [18], with less adverse effects [19] and that can potentially change HIV-related behaviours, reduce crime and improve quality of life [20-25]. As a first step in this advocacy process, the findings of this evaluation were reported to the joint meeting of the NACO and the partner NGOs working with IDUs in India with funding support from DFID held on the November 17–18, 2006 at New Delhi. What has been established needs to be continued as well as expanded in future.

## Abbreviations

AIDS: Acquired Immune Deficiency Syndrome; ART: Antiretroviral Treatment; DFID: Department for International Development; DIC: Drop-in-centre; EHA: Emmanuel Hospital Association; FGD: Focus group discussion; HIV: Human immunodeficiency virus; IDU: Injecting drug user; NACO: National AIDS Control Organization; NGO: Non-governmental organization; OST: Opioid substitution treatment; STI: Sexually transmitted infections; TB: Tuberculosis; UNODC ROSA: United Nations Office on Drugs and Crime Regional Office for South Asia; VCCT: Voluntary confidential counselling and testing; WHO: World Health Organization.

## Competing interests

The authors declare that they have no competing interests.

## Authors' contributions

The evaluation of the treatment centres in Manipur and Nagaland were carried out by MSK and RDN respectively. MSK drafted the manuscript and incorporated all suggestions from the coauthors. All coauthors made significant contributions to the interpretation of the data and drafting of the manuscript, and they all approved the version submitted.
